# Optimizing Virtual Follow-Up Care: Realist Evaluation of Experiences and Perspectives of Patients With Breast and Prostate Cancer

**DOI:** 10.2196/65148

**Published:** 2025-01-03

**Authors:** Sarah Scruton, Geoff Wong, Stephanie Babinski, Lauren R Squires, Alejandro Berlin, Julie Easley, Sharon McGee, Ken Noel, Danielle Rodin, Jonathan Sussman, Robin Urquhart, Jacqueline L Bender

**Affiliations:** 1 Cancer Rehabilitation and Survivorship Department of Supportive Care Princess Margaret Cancer Centre Toronto, ON Canada; 2 Nuffield Department of Primary Care Health Sciences University of Oxford Oxford United Kingdom; 3 Dalla Lana School of Public Health University of Toronto Toronto, ON Canada; 4 Department of Radiation Oncology University of Toronto Toronto, ON Canada; 5 Radiation Medicine Program Princess Margaret Cancer Centre Toronto, ON Canada; 6 TECHNA Institute University Health Network Toronto, ON Canada; 7 Department of Medical Education Dr Everett Chalmers Regional Hospital Horizon Health Network Fredericton, NB Canada; 8 Department of Medicine Division of Medical Oncology The Ottawa Hospital and the University of Ottawa Ottawa, ON Canada; 9 The Walnut Foundation Brampton, ON Canada; 10 Department of Oncology McMaster University Hamilton, ON Canada; 11 Department of Community Health and Epidemiology Dalhousie University Halifax, ON Canada; 12 Institute of Health Policy Management and Evaluation University of Toronto Toronto, ON Canada

**Keywords:** cancer, follow-up, virtual, outcomes, realist evaluation, survivorship

## Abstract

**Background:**

Virtual follow-up (VFU) has the potential to enhance cancer survivorship care. However, a greater understanding is needed of how VFU can be optimized.

**Objective:**

This study aims to examine how, for whom, and in what contexts VFU works for cancer survivorship care.

**Methods:**

We conducted a realist evaluation of VFU among patients with breast cancer and prostate cancer at an urban cancer center during the COVID-19 pandemic. Realist evaluations examine how underlying causal processes of an intervention (mechanisms) in specific circumstances (contexts) interact to produce results (outcomes). Semistructured interviews were conducted with a purposive sample of patients ≤5 years after diagnosis. Interviews were audio-recorded and analyzed using a realist logic of analysis.

**Results:**

Participants (N=24; n=12, 50% with breast cancer and n=12, 50% with prostate cancer) had an average age of 59.6 (SD 10.7) years. Most participants (20/24, 83%) were satisfied with VFU and wanted VFU options to continue after the COVID-19 pandemic. However, VFU impacted patient perceptions of the quality of their care, particularly in terms of its effectiveness and patient centeredness. Whether VFU worked well for patients depended on patient factors (eg, needs, psychosocial well-being, and technological competence), care provider factors (eg, socioemotional behaviors and technological competence), and virtual care system factors (eg, modality, functionality, usability, virtual process of care, and communication workflows). Key mechanisms that interacted with contexts to produce positive outcomes (eg, satisfaction) were visual cues, effective and empathetic communication, and a trusting relationship with their provider.

**Conclusions:**

Patients value VFU; however, VFU is not working as well as it could for patients. To optimize VFU, it is critical to consider contexts and mechanisms that impact patient perceptions of the patient centeredness and effectiveness of their care. Offering patients the choice of in-person, telephone, or video visits when possible, coupled with streamlined access to in-person care when required, is important. Prioritizing and addressing patient needs; enhancing physician virtual socioemotional behaviors and technology competency; and enhancing VFU functionality, usability, and processes of care and communication workflows will improve patient perceptions of the patient centeredness and effectiveness of virtual care.

## Introduction

According to report “Canadian Cancer Statistics 2023,” 1 in 2 Canadians will be diagnosed with cancer in their lifetime, and two-thirds of Canadians diagnosed with cancer today will live for ≥5 years after their diagnosis [1]. Among the most common cancers in men and women, the 5-year net survival rate is 87% for breast cancer (BC) and 95% for prostate cancer (PC) [1]. While these outcomes reflect advances in early detection and treatment, they have substantially increased the demand for follow-up care services. Optimal follow-up care consists of surveillance for cancer spread, recurrences, or secondary cancers; prevention and management of acute and persistent treatment side effects; and promotion of healthy behaviors to mitigate new and ongoing health concerns [2]. To be effective, this involves ongoing assessment and comprehensive care, including referral to specialized supportive care services to improve quality of life, reduce disability, and restore function [3,4].

In Canada, and internationally, follow-up cancer care is typically provided by oncologists in outpatient secondary or tertiary care settings. Evidence suggests that this model of in-person follow-up care with a specialist is not sustainable and is not working well for patients [5]. Oncology offices are often overcrowded with long wait times, short appointments, and high costs per visit [6]. Furthermore, the high burden of unmet posttreatment supportive care needs suggests that the existing model of follow-up care is not meeting patients’ needs [7,8]. Alternatives such as follow-up with primary care providers are comparable to oncologist-led follow-up in terms of detection of recurrence, survival, and quality of life [9,10]. However, prior work [6,11,12] shows that many survivors of cancer prefer to be seen by an oncologist for follow-up due to concerns about the quality and continuity of care.

One method proposed to improve the experience and effectiveness of cancer follow-up care is virtual care, defined as remote interactions between patients and care providers using technology (ie, video calls, telephone, email, etc) [13]. Virtual follow-up (VFU) could alleviate pressure on clinicians and health care services and better meet patient needs by providing patients with access to timely, convenient, and tailored follow-up care [14]. Before the COVID-19 pandemic, virtual care was infrequently used in Canada, mainly to provide care to rural and remote populations [13], and the evidence of its effectiveness was limited [15]. During the COVID-19 pandemic, there was rapid and widespread adoption of virtual care in Canada and globally [16]. In a prior study, we demonstrated that most patients with cancer were satisfied with VFU during the COVID-19 pandemic, but some did not want VFU to continue after the COVID-19 pandemic [17]. In addition, while virtual visits made care more accessible for some, other studies have shown that virtual care has exacerbated access disparities by widening the digital divide for those who lack the knowledge or technological means to access care virtually [18,19].

To sustain VFU as an option for patients with cancer, we need a better understanding from patients on how, for whom, and in what contexts this model of care works. Patient perspectives on how to optimize VFU care can inform updates to telehealth clinical practice guidelines, such as those offered by the American Association of Clinical Oncology (ASCO) early in the COVID-19 pandemic [20], to ensure patient-centered virtual care. Realist evaluations are ideally suited to answer these questions. Realist evaluations are a proven, theory-driven method that enable a rigorous understanding of context and its influence on causal processes (ie, mechanisms) to explain how and for whom an innovation works best [21,22]. This method is increasingly used to assess complex innovations in health care, particularly those that involve multiple interconnected components and are dependent on individual responses and the wider context for their success [23,24]. Therefore, we conducted a realist evaluation [21] to examine how, for whom, and in what contexts VFU works for patients with BC and PC and conclude with recommendations for optimizing VFU for patients with cancer.

## Methods

### Ethical Considerations

The study was approved by the University Health Network Institutional Research Ethics Board (ID 21-5397). Informed consent was obtained from all study participants before data collection, and all data were deidentified before analysis. All study participants received a CAD $25 honorarium to compensate for their time. At the time of the study, US $1 was equal to CAD $1.30.

### Study Design

We conducted a realist evaluation following the steps by Pawson and Tilley [21] and RAMESES [23] quality and reporting guidelines.

Step 1 involved developing an initial program theory, which is a kind of conceptual framework, to explain how VFU could be delivered to work most effectively. This involved explaining how the underlying processes of VFU care (mechanisms) interact with specific circumstances (contexts) to produce results (outcomes). For this step, we used prior evidence to understand patient views [25] and the effectiveness of virtual versus in-person cancer care [15], theories from technology adoption [26-28] to understand factors that influence the adoption of VFU and theories from patient-provider communication [29] to determine the essential elements of effective clinical encounters, and the 6 domains of health care quality identified by the Agency for Healthcare Research and Quality [30] to understand the attributes of quality VFU. Using these information sources, we created a figure to illustrate how VFU can bring about the desired outcomes. We then held a 2-hour virtual workshop with project team members and stakeholders, consisting of clinicians, researchers, and patient partners (N=10) to obtain their views on the initial theory.

Step 2 involved collecting evidence on the contexts, mechanisms, and outcomes of VFU. For this, we (1) administered a web-based survey to patients at a cancer centre to obtain information on their sociodemographic characteristics, experience, and satisfaction with VFU [17]; (2) purposively recruited a subsample of survey respondents to participate in semistructured interviews to explore their views and experiences with VFU in greater depth; and (3) administered a short follow-up survey to collect information on interview participants’ psychosocial well-being.

Steps 3 and 4 involved data integration and synthesis using a realist logic of analysis. The goal of this analysis was to determine whether data were functioning as context, mechanism, or outcome, and if so, within which context-mechanism-outcome configuration (CMOC) [23]. We then held a 2-hour virtual stakeholder workshop with a wider group of stakeholders (N=12) to obtain feedback on the relevance of our findings more broadly.

### Setting and Participants

This study was conducted at the Princess Margaret (PM) Cancer Centre, a tertiary, university-affiliated, teaching hospital, which is a part of the University Health Network in Toronto, Canada. Before COVID-19, <1% of visits at PM were conducted virtually [31]. After the declaration of the COVID-19 pandemic, 68% of visits at PM were virtual [31]. The participants in this study comprised a subsample of patients with BC and PC who had completed the PM Virtual Care Evaluation Survey between May and July 2021 [17] and who agreed to participate in a follow-up interview. Interviews occurred between September and November 2021. Survey participants had to meet the following criteria: they must have received a cancer diagnosis, be aged >18 years, have participated in at least 1 virtual appointment (eg, by phone or video) in the last 12 months, and have a valid email address on file. We used the survey data to intentionally select interview participants who varied in age, ethnicity, geographic location, and satisfaction with VFU. In addition, to be eligible to participate in an interview, survey respondents must have agreed to be contacted about a follow-up interview, received a diagnosis of BC or PC within the last 5 years, and completed treatment for cancer or were receiving adjuvant hormone therapy. On the basis of previous research, a sample size of 15 per patient group (30 in total) was estimated to be sufficient [32,33]. However, data collection continued until theoretical saturation [34].

### Data Collection

First, data on participants’ sociodemographic characteristics and virtual care use and perceptions were extracted from the PM Virtual Care Evaluation Survey to describe the sample and inform each interview. Demographic data included age, whether they were born in Canada (yes or no), English as a first language (yes or no), race or ethnicity (10 response options), highest level of education (high school or less, college or technical school, university undergraduate, or postgraduate), and household income (CAD <$60,000, CAD $60,000-$99,000, or CAD ≥$100,000). Virtual care use and preferences were assessed by asking participants to indicate the type of virtual appointments they had received and would have liked to receive in the past 12 months (phone, video, or both). Patient satisfaction with virtual care was captured using a 5-point Likert response (very satisfied, satisfied, neutral, dissatisfied, or very dissatisfied). Patient desire for virtual care options after the COVID-19 pandemic was captured by asking participants to indicate their level of agreement with the statement “I would like to continue to have virtual options for some of my visits after the COVID-19 pandemic ends,” using a 5-point Likert response option (strongly agree, agree, neutral, disagree, or strongly disagree).

Each participant then took part in one 45- to 60-minute semistructured interview based on the realist approach [21,22]. They were asked about their initial expectations and desired outcomes of VFU, how it worked or did not work for them, and how VFU could be made to work optimally. The interviews were conducted by a research coordinator with experience in qualitative interview methods (SB) who was trained in realist methodology by a realist expert on the team (GW). Interviews were audio-recorded and transcribed verbatim.

After the interview, participants completed a brief survey on their level of patient activation, anxiety, and depression. Patient activation was measured using the Patient Activation Measure [35], which scores a patient’s knowledge, skills, and confidence for proactively managing their health and health care using a 13-item tool with 4-point Likert scales. The Patient Activation Measure scores (between 0 and 100) were converted into four levels of activation: (1) not believing (patient) activation is important (score of ≤47.0), (2) a lack of knowledge and confidence (score of 47.1-55.1), (3) beginning to take action (score of 55.2-67.0), and (4) taking action (score of ≥67.1). Anxiety was measured using the Generalized Anxiety Disorder 7-item [36], and depression was measured using the Patient Health Questionnaire-9 [37]. Anxiety and depression scores were converted into the following categories: minimal (score of 0-4), mild (score of 5-9), moderate (score of 10-14), moderately severe (depression score only [15-19]), and severe (score of 20-27 on Patient Health Questionnaire-9 or 15-21 on Generalized Anxiety Disorder 7-item). Fear of recurrence was measured using the Fear of Cancer Recurrence Inventory-Short Form [38], a 9-item severity scale that evaluates intrusive thoughts associated with fear of cancer recurrence using 4-point Likert scales. A score of ≥13 on the Fear of Cancer Recurrence Inventory-Short Form denotes a clinically relevant fear of cancer recurrence.

### Data Analysis

A realist logic of analysis was used to analyze interview transcripts [21], with the support of NVivo (version 14; Lumivero). The coding was both deductive (some codes were created in advance informed by the initial program theory) and inductive (some codes emerged from the data). The data were initially sorted into broad conceptual categories or “buckets” by 2 team members independently (SB and SS) through consultation with the lead author (JLB). The buckets were developed following data collection both deductively and inductively based on the initial program theory and what was found in the data. They were further analyzed by 1 team member (SS) to develop preliminary themes and CMOCs. As there were no meaningful differences across the 2 groups of BC and PC participants, the data were analyzed and reported jointly. The themes and CMOCs underwent an intensive refinement process with 3 research team members (JLB, GW, and SS) to ensure they were clear, concise, and representative of the interview data. Repetitive or redundant CMOCs were removed. The data were then used to refine the program theory. Descriptive statistics were used to summarize patient demographic characteristics, virtual care use, patient activation, and psychosocial well-being using R (version 4.1.2; R Foundation for Statistical Computing).

## Results

### Participant Characteristics

In total, 24 patients were interviewed; of these, 12 (50%) had been diagnosed and treated for BC and 12 (50%) had been diagnosed and treated for PC (Table 1). On average, the participants were aged 65.9 (SD 8.65) years, 100% (24/24) attended postsecondary school, and 71% (17/24) reported a household income of CAD ≥$100,000. Approximately half (11/24, 46%) of the sample identified as racialized, 50% (12/24) were born outside of Canada, and 20% (5/24) had a non-English first language. The most common treatment type was surgery (18/24, 75%), followed by radiation therapy (17/24, 71%) and chemotherapy (9/24, 37%). This was a highly activated sample, with most (18/24, 75%) at level 3 or 4 on the patient activation scale and most with relatively mild or minimal depression (18/24, 75%) or anxiety (20/24, 83%), and most participants (15/24, 63%) reported nonclinical levels of fear of recurrence.

**Table 1:** Participant characteristics (N=24).

Characteristic					Prostate cancer (n=12)			Breast cancer (n=12)			Total
**Demographic characteristics**											
	Age (y), mean (SD)			65.92 (8.65)			53.25 (14.31)			59.58 (13.41)	
	**Race or ethnicity, n (%)**										
		Arab or West Asian	0 (0)			1 (8)			1 (4)		
		Black	1 (8)			2 (17)			3 (13)		
		East Asian	0 (0)			1 (8)			1 (4)		
		Jewish	1 (8)			0 (0)			1 (4)		
		Latin American	0 (0)			2 (17)			2 (8)		
		Southeast Asian	0 (0)			2 (17)			2 (8)		
		South Asian	1 (8)			0 (0)			1 (4)		
		White	9 (75)			4 (33)			13 (54)		
	**Born in Canada** **, n (%)**										
		Yes	5 (42)			7 (58)			12 (50)		
	**English as a first language, n (%)**										
		Yes	10 (83)			9 (75)			19 (79)		
	**Highest level of education received, n (%)**										
		High school or less	0 (0)			0 (0)			0 (0)		
		Postsecondary school	12 (100)			12 (100)			24 (100)		
	**Household income (CAD $1.30=US $1), n (%)**										
		60,000-99,999	2 (17)			2 (17)			4 (17)		
		≥100,000	8 (67)			9 (75)			17 (71)		
		Prefer not to say	2 (17)			1 (8)			3 (12)		
**Clinical and psychosocial characteristics**											
	**Treatments received, n (%)**										
		Drug or chemotherapy	0 (0)			9 (75)			9 (37)		
		Hormone therapy	1 (8)			5 (42)			6 (25)		
		Radiation therapy	6 (50)			11 (92)			17 (71)		
		Surgery	8 (67)			10 (83)			18 (75)		
		Other	1 (8)			0 (0)			1 (4)		
	**Anxiety level** **, n (%)**										
		Minimal or mild	10 (83)			10 (83)			20 (83)		
		Moderate or severe	1 (8)			2 (17)			3 (13)		
	**Depression level, n (%)**										
		Minimal or mild	11 (92)			7 (58)			18 (75)		
		Moderate, moderately severe, or severe	1 (8)			4 (33)			5 (21)		
	**Level of patient activation** **, n (%)**										
		Not believing activation is important	2 (17)			1 (8)			3 (13)		
		A lack of knowledge or confidence	0 (0)			1 (8)			1 (4)		
		Beginning to take action	5 (42)			4 (33)			9 (37)		
		Taking action	4 (33)			5 (42)			9 (37)		
	**Fear of recurrence, n (%)**										
		Clinical	4 (33)			3 (25)			7 (29)		
		Nonclinical	7 (58)			8 (67)			15 (63)		
		Missing	1 (8)			1 (8)			2 (8)		

### VFU Use and Preferences

All participants received a phone call appointment (Table 2). Most patients with BC also received a video appointment (20/24, 83%); none of the patients with PC received a video appointment. Overall, most participants (20/24, 83%) were satisfied or highly satisfied with their virtual care visits, and most participants (20/24, 83%) would want VFU options to continue after the COVID-19 pandemic. When asked which type of VFU appointment they would prefer in the future, most patients (15/24, 63%) indicated video appointments.

**Table 2:** Virtual follow-up (VFU) characteristics (N=24).

Characteristic			Prostate cancer (n=12)		Breast cancer (n=12)		All
**Type of VFU appointment received, n (%)**							
	Only phone	12 (100)		2 (17)		14 (58)	
	Only video	0 (0)		0 (0)		0 (0)	
	Phone+video	0 (0)		10 (83)		10 (42)	
**Satisfaction with virtual care, n (%)**							
	Very satisfied	7 (58)		7 (58)		14 (58)	
	Satisfied	4 (33)		2 (17)		6 (25)	
	Dissatisfied	1 (8)		3 (25)		4 (17)	
**Would want the option for VFU after the COVID-19 pandemic, n (%)**							
	Strongly agree	6 (50)		7 (58)		13 (54)	
	Agree	4 (33)		3 (25)		7 (29)	
	Neutral	2 (17)		2 (17)		4 (17)	
	Disagree	0 (0)		0 (0)		0 (0)	
**Preferences for virtual care in the future, n (%)**							
	No preference	1 (8)		4 (33)		5 (21)	
	Phone	4 (33)		0 (0)		4 (17)	
	Video	7 (58)		8 (67)		15 (63)	

### Views and Experiences With VFU

Overview

**Figure 1:**
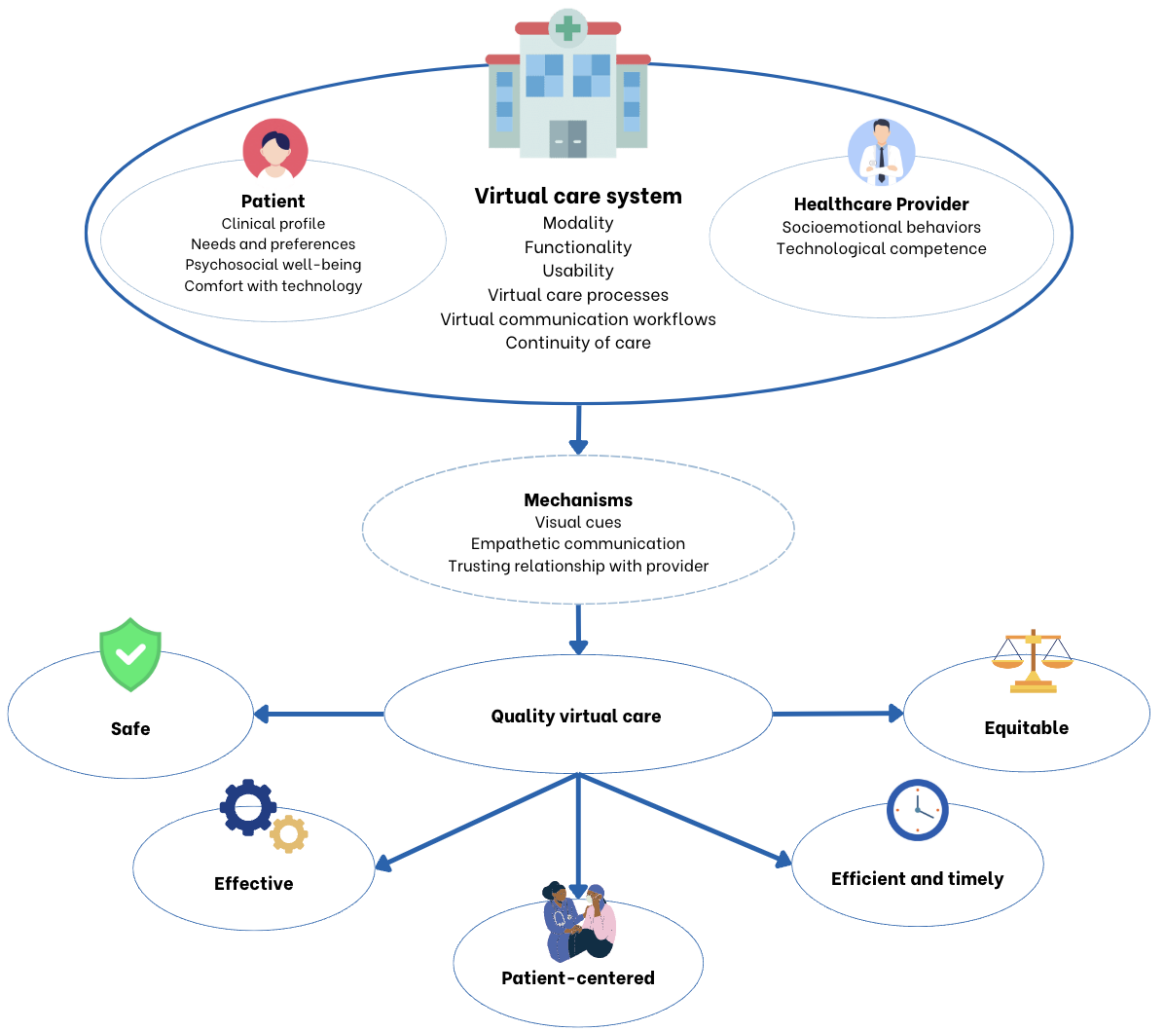
Refined program theory.

We identified 8 overarching concepts that captured patients’ views on how VFU could be made to work effectively. These 8 concepts consisted of 24 themes and 46 CMOCs (Multimedia Appendix 1). Due to the number of CMOCs that were developed, each CMOC is labeled with a code (eg, A1, A2) found in Multimedia Appendix 1 (for ease of reference). The findings related to each CMOC are labelled with a matching code throughout the Results section. The concepts, themes, and CMOCs were used to refine the program theory (Figure 1).

#### Effectiveness

Effectiveness of VFU appears to be influenced by patient-, physician-, and disease-related contextual factors. For example, VFU was considered ineffective if it replaced physical appointments all together, as physical examinations and speaking to a physician in person reduced patient anxiety about recurrence and management of ongoing side effects (A1). In-person appointments provided visual cues (ie, body language, facial expression, etc) that helped patients understand the meaning of information received from their provider and feel more confident in their care (A2). However, participants felt that this may be partly overcome through video appointments, as video allows patients and care providers to read each other’s body language and facial expressions. This may be particularly important for patients experiencing high levels of anxiety about their cancer, as the lack of visual cues with phone calls causes VFU to be insufficiently reassuring (A3). Participants also felt that it was important for physicians to see the visual cues of the patient, as this would enable the physician to pick up on distress or concern that may not be verbally expressed by patients and act accordingly (A4). The final contextual factor described to impact effectiveness of VFU was the perceived loss of the appreciation of the seriousness of cancer. In-person appointments act as a reminder of the seriousness of the condition. Without in-person appointments, patients were concerned that they may become less vigilant in their self-care and self-management (eg, nonadherence to medication or forgetting self-examinations; A5).

#### Efficiency and Timeliness

If participants felt that the VFU they received was both efficient and timely, they were more likely to be satisfied with their care. These participants felt that VFU would likely improve the overall efficiency of follow-up care, as they believed hospitals could save time and money by scheduling more appointments per day (B1). Participants also believed that greater use of virtual appointments could improve the experience of in-person appointments (when required), as hospital waiting rooms would be less crowded and stressful (B2).

However, some participants expressed concerns that VFU could lead to delays in receiving care. Many described being given very long windows of time for their VFU appointments, which were difficult and frustrating to plan around (B3). In some cases, physicians called outside of these time windows, which led to missed appointments or patients having to speak to someone other than their principal care provider (B4). These types of situations were anxiety inducing for patients, particularly if they had concerns to share with their provider as they feared that their condition would worsen while waiting for a rescheduled appointment (B5). Issues with timeliness may also occur if patients have difficulty booking timely appointments due to long wait times for callbacks or uncertainty about whom to contact (B3).

#### Patient Centeredness

The largest conceptual bucket by far was related to patient centeredness. Participants described ways in which VFU can be more or less patient-centered compared to in-person care, and how this impacted how well VFU worked for them. This included references to personal values, preferences, and expectations of VFU and how these measured up to their experiences. A patient’s preferences and expectations were influenced not only by their unique life circumstances but also by health system characteristics, such as continuity of care. Participants were concerned that the number of care providers involved in their care may increase with VFU due to physicians being busier with more appointments. Participants explained that receiving care from multiple care providers made them feel less cared for because they had less time to form trusting relationships with each provider (C1). This was particularly anxiety inducing for those who were newly diagnosed, as they were particularly vulnerable and in need of a strong physician-patient relationship (C2). In addition, they explained that receiving care by multiple care providers led to poor continuity and quality of care due to a lack of communication between care providers. This was frustrating for participants, as they felt they had to continually repeat their history to their care providers, and they were concerned that their care would be negatively impacted by their physician not having a comprehensive understanding of their case (C3 and C4).

Difficulty forming relationships with care providers was also heightened when participants had never been given the opportunity to meet their provider face-to-face, as they felt this hindered their ability to open up emotionally (C5). Some participants felt that the care they received was more holistic and empathetic when provided face-to-face, and they valued the ability to visually see that they had the full attention of their care provider (C6). Therefore, participants appreciated when their care providers would make use of video calls for their VFU appointments. However, most participants also expressed that their VFU appointments felt rushed compared to in-person appointments, which made them feel less cared for as they were unable to take the time needed to process information and ask questions (C7). Many participants felt their VFU appointments only addressed their clinical or medical issues, as opposed to the personal issues that accompany a cancer diagnosis, which caused them to have unmet needs (C8). This may be because the nature of VFU tends to favor information provision rather than emotional support (C9).

When patients leave active treatment, some described feeling “lost in the system.” This may be increased with VFU, as not entering hospitals means that it may be more difficult for patients to access support when needed (C10). Participants felt that VFU may be improved if it included straightforward processes to obtain information, support, and care when needed, which would prevent them from feeling abandoned. This could include the ability to communicate with their care provider via technology (ie, email) in between appointments (C11 and C12). In addition, participants expected their care providers to continue to provide referrals to supportive care programs, but this was not always the case with VFU (C13).

Finally, participants appreciated that VFU improved the convenience of care. Patients did not have to travel to the hospital for their appointment, which saved them time and money (C14). They also appreciated that they could attend their appointments from anywhere, including from the comfort of their own home, which avoided stressful and slow waiting rooms (C15). This also meant they could multitask while waiting for appointments, meaning VFU was less disruptive to their schedule (C15). VFU also allowed family members to be included in VFU appointments, which was particularly valuable when COVID-19 prevented family members from attending in-person appointments (C16). Many participants felt that these advantages of VFU outweighed the benefits of in-person care.

#### Equity

Follow-up care works best when it is equitable for every patient. Participants noted that VFU may limit access to care for those with certain sociodemographic characteristics such as a disability, older age, or low socioeconomic status. For example, participants expressed concern that someone with hearing loss may face difficulties speaking over the phone, and someone with vision loss may struggle with video appointments (D1). Some participants also considered that some individuals may not have access to fast internet or that those with a lower level of education may be unable to search for medical information on the internet if they are not given time to ask questions during VFU appointments (D2 and D3).

#### Safety

Participants noted that VFU could improve safety by decreasing their risk of being exposed to infectious diseases such as COVID-19 (E2). However, some participants felt that VFU may be less safe because it removed the ability to be physically examined by an oncologist, potentially resulting in poorer care. Turning elsewhere for care (such as the emergency room) caused participants to feel they were being inadequately cared for (E1).

#### Patient Characteristics

Whether VFU worked well depended on patient characteristics that impacted their suitability and preferences for virtual care. This included comfort with technology, as some participants described challenges that they and others may have with navigating and using VFU technology (F1). Participants who had experience working within a virtual environment had an easier time using VFU and therefore were more likely to be satisfied with it (F2).

Other patient factors that influenced whether VFU was suitable included clinical characteristics such as mental health, disease characteristics, or comorbidities. Those with complex issues (ie, fear of recurrence, severe side effects, anxiety, and recently completed treatment) reported a greater need for reassurance, acknowledgment, and help coping with their adversities, along with a higher reliance on physical examinations, making them a poorer candidate for VFU (F3). The opposite was true for those who experienced fewer side effects and whose appointments mainly addressed simple routine check-ups, making them ideal candidates for VFU (F4).

At the same time, VFU seemed to overcome access barriers experienced by those who are less likely to seek health care. Participants who described themselves as being reluctant to access the health care system (eg, due to a lack of time, downplaying their condition, not the type to seek care, etc) appreciated the convenience and efficiency of VFU, which they explained motivated them to access health care (F5). Finally, a patient’s preference for VFU seemed to depend on their coping style. Those who described themselves as requiring considerable emotional support (ie, emotional coping) felt that VFU did not work as well for them because VFU tends to focus on information provision over emotional support (F6). In contrast, those who did not require as much emotional support (ie, problem-focused coping), appreciated the simple, quick information-provision appointments (F7).

#### Physician Characteristics

Many participants described the importance of physician competence and bedside manner regardless of whether the appointment was virtual or in person. If a physician did not appear to be comfortable with the VFU technology, participants judged the physician as less competent in providing care and were concerned that this could impact the quality of their care (G1). In addition, if a physician was less empathetic, patient, or comforting during VFU compared to in-person appointments, participants stated that they would not prefer this type of care as they would feel less cared for (G2). Most participants described feeling more rushed and less listened to during phone appointments, as if the appointments were only for the purpose of sharing information such as test results and not for the purpose of addressing emotional concerns (G3). Some participants felt that “webside manner” is something that could be improved with proper training (G4).

#### Virtual Care System Characteristics

The final factor found to impact whether VFU works well for patients was the characteristics of the virtual care system itself. The most discussed topic was VFU modality, as most patients were not offered the choice between having their VFU appointments via phone or video. Some participants were okay with this, as they felt the VFU modality that they were offered was effective for their needs. However, others were disappointed that they were not given a choice in the modality. They felt that being provided the option for video appointments would have increased the quality of their care as they could have benefitted from face-to-face communication (H1). That said, this was only true if the VFU technology worked well (functionality) and was easy to use (usability). Some participants were frustrated with the VFU technology because it was difficult for them to use and did not work well for them (H2). However, when VFU technology does work well, participants described being appreciative of the fact that they could easily access appointments and test results and communicate with their care providers. Participants felt that physicians should be responsible for offering fallback plans if the VFU technology did not work well, such as what to do when a call drops, so that they need not be fearful of the technology malfunctioning (H3). Some participants noted the lack of virtual care processes and communication channels (virtual communication workflows). This caused issues when they did not know how to contact support staff and schedule appointments, when they did not know if their appointment would occur outside of the scheduled time window, and when they did not know what to do if their VFU call or internet connection was dropped or the technology malfunctioned (H4).

### Recommendations

The study findings point to several strategies that health care providers and health care systems can use to enhance the patient centeredness and perceived effectiveness of VFU appointments for patients. We have outlined these patient-derived recommendations in Textbox 1, which align with ASCO’s guidelines for telehealth in oncology [38] and provide additional considerations to optimize VFU for patients.

**Patient-derived recommendations for improving virtual follow-up (VFU) care.**

1. Offer patients the choice of an in-person or virtual visit
2. Offer patients the choice of virtual visit modality (eg, telephone or video)
3. Ensure there is an easy-to-use and efficient process for patients to schedule an in-person visit if needed
4. Clearly communicate how the appointment can be continued if the technology fails and provide technical support
5. Ensure that virtual visit booking and scheduling processes are flexible enough to cater to patient’s changing needs and expectations and reduce wait times where possible
6. Make time to listen to patient concerns and provide emotional support during virtual visits
7. Demonstrate empathy and provide your full attention using active listening skills during virtual visits
8. Demonstrate competency with virtual care technology
9. Ensure patients are provided resources and referrals to survivorship care programs during or after a virtual visit
10. Improve the quality of VFU technology and work flows to create a seamless and reliable care experience for patients

## Discussion

### Principal Findings

To our knowledge, this is the first realist evaluation of VFU for cancer care. This study has helped to answer for whom and in which contexts VFU is most suitable and has identified ways in which VFU could be optimized to better meet the needs of patients with cancer receiving follow-up care. Overall, the patients with BC and PC in this study described themselves as being satisfied with VFU during the COVID-19 pandemic and wanted VFU options to continue after the COVID-19 pandemic. However, VFU impacted patient perceptions of the quality of their care, particularly in terms of its effectiveness and patient centeredness. Whether VFU worked well for patients depended on patient factors (eg, needs, psychosocial well-being, and technological competence), provider factors (eg, socioemotional behaviors and technological competence), and virtual care system factors (eg, modality, functionality, usability, and virtual processes of care and communication workflows). Key mechanisms that interacted with contexts to produce positive outcomes (eg, satisfaction and reassurance) were visual cues, clear empathetic communication, and a trusting relationship with their provider.

### Is Virtual Care Suitable for All Cancer Follow-Up Appointments?

While follow-up appointments have been identified by patients with cancer and health care providers and recommended by clinical practice guidelines as suitable for virtual care delivery because of their relative brevity and simplicity [20,39,40], they can be anxiety provoking and complex depending on the circumstances. For example, moderate to severe “scanxiety” (the fear, stress, and anxiety in anticipation of surveillance tests in follow-up cancer care) is present in as many as 28% of survivors of cancer [41]. In a prior study, we reported that survivors of cancer who experienced distress (anxiety or depression) were less likely to be satisfied with VFU and less likely to want VFU in the future compared to those who did not report distress [17]. This study has revealed that patients experiencing distress may not be satisfied with VFU because the quality of emotional support provided virtually is less effective. Participants felt more comforted during in-person visits because they had more time to discuss their concerns and the type of emotional support received was more effective because the visual cues of the provider conveyed empathy and understanding. Interestingly, a systematic review comparing virtual to in-person cancer care on the psychosocial outcomes of patients with cancer found that virtual care from physicians or nurses was more beneficial for patients during active treatment than follow-up [42]. However, a systematic review comparing virtual versus in-person cancer care found virtual psychosocial counseling to be equally effective for follow-up care [15]. The ASCO telehealth guidelines recommend providing individualized orientation and instruction to patients and care providers on the specific technology that will be used for the virtual care interaction [20]. Our findings highlight a need for care provider training in empathetic virtual communication as well, which has been expressed by care providers themselves [43], accompanied with seamless referrals to psychosocial professionals for follow-up psychosocial issues.

### For Whom and in Which Contexts VFU Is Most Suited

Importantly, this study has identified for whom and in which contexts VFU is most suited—a knowledge gap that has been identified by many [39,44,45]. In doing so, the study findings align with and considerably expand upon the recommendations of the European Society of Medical Oncology on which patients should be offered telehealth [46]. In its current form, this study has found that VFU is more suited for patients with cancer who are doing well—physically and emotionally—often termed “the well follow-up patient” [11]. From a clinical perspective, this includes patients with no evidence of disease; with limited or well managed treatment side effects including no-to-mild depression, anxiety, and fear of recurrence; who have problem-focused coping styles; and who require limited emotional support from professionals to deal with the effects of cancer and its treatment. From a sociodemographic perspective, this also includes patients who are comfortable speaking in English (for appointments that are in English); who do not have auditory, visual, cognitive, or physical impairments that limit their effective use of VFU technology; who have access to and experience with VFU technology; and who have the education and skills needed to proactively manage their health and health care. VFU is also well suited for patients with cancer with busy schedules and many competing demands, as they appreciate the convenience of virtual visits. It may even increase health care access for those who are typically reluctant to seek care due to the inconvenience of in-person visits.

Hence, it its current form, if VFU was the only option, it would likely increase disparities in access to care for some patients with cancer. Other studies have demonstrated this to be the case. Notably, a study of health care use during the COVID-19 pandemic in the United States found that Black patients had 0.6 times of the adjusted odds of accessing care through telemedicine compared to White patients [19]. However, subgroup analysis revealed that younger Black women were more likely to access care through telemedicine, particularly for urgent care issues. In contrast, a study of access disparities conducted at the same institution as this study identified no differences in virtual care use based on patient demographics [47]. This may be due to the higher socioeconomic status of their study sample, as only 7% identified as low income. Importantly, they found that regardless of visit type, patients who were structurally marginalized by ethnocultural, situational, and residential status, as well as gender, were less satisfied with their care whether it was virtual or in person. These findings highlight the need for proactive and continuous efforts to identify and intervene to address health disparities in access, experience, and outcomes.

### How Health Care Providers Can Optimize Virtual Care for Patients

This study also identified strategies that care providers can use to enhance the patient centeredness and perceived effectiveness of VFU for patients. In fact, health care provider behavior was the key contextual factor that triggered the mechanisms (eg, assessment of visual cues, providing full attention, etc) that led to positive outcomes (eg, patient responses such as trust, reassurance, feeling cared for, and satisfaction). VFU was considered ineffective for patients if care providers did not give patients the option of choosing in-person versus virtual appointments; were not competent using virtual care technology; did not give patients their full attention and time; were less empathetic, patient, and comforting during the virtual visit; or focused on collecting or providing information at the expense of emotional support. Collectively, except for technological competence, these behaviors reflect socioemotional behaviors critical for the effectiveness of the patient-physician encounter. Drawing on social interaction theory, Roberts and Aruguete [48] have shown that patients mostly recognize and react to physician socioemotional behaviors (eg, concern, affection, and attention displayed verbally and nonverbally) due to a lack of understanding of physician task behaviors (eg, explanation of etiology, symptoms, and treatment). In a series of experiments using videotapes of varied levels of physician task versus socioemotional behavior, they demonstrated that high levels of socioemotional behaviors increased patient self-disclosure, trust, satisfaction, and likelihood of recommending the physician. Participants in this study recognized that physician “webside manner” could be improved through training. Likewise, a study of key informants representing Canadian provincial and national health care organizations with expertise in virtual care delivery [43] identified a need to train care providers in how to use technology and integrate virtual care into practice, adapt their clinical skills (including examination skills) to virtual care, build rapport through good communication with patients, and understand when an in-person visit is necessary based on the nature of the appointment and patient contextual factors.

### How Health Care Systems Can Optimize Virtual Care for Patients

System transformation is needed to deliver virtual care optimally and equitably to patients. First, health care systems need to enhance virtual care infrastructure and workflows for virtual care to realize its potential for greater efficiency. From a patient perspective, this includes ensuring that VFU technology is accessible, easy to use, and reliable. To increase accessibility, high technology (eg, video) and low technology (telephone and text) VFU solutions are required. Most Canadian studies of virtual care during the COVID-19 pandemic [17,30,49], including this study, reported greater use of telephone than video for virtual care. This was likely due to limited virtual care infrastructure, problems encountered using video, and limited video technology proficiency of patients and care providers. As virtual care technology innovates, low technology options should remain and be offered to patients to ensure equitable access, as broadband connectivity remains inconsistent across Canada [50]. However, the virtual care reimbursement structure needs to change to adequately reimburse care providers for telehealth services. Currently, Ontario’s virtual care funding model limits telephone-based care by compensating physicians less for telephone visits compared to in-person visits or video visits [51]. Consequently, physicians might opt to limit their use of telephone visits, potentially leading to reduced access to care for those without high-speed internet access. Establishment of virtual care processes and workflows, along with patient education materials, are needed to seamlessly navigate patients through their virtual care appointment. From a patient perspective, this includes ensuring patients are given the option to choose their virtual visit modality, informed how to use VFU technology optimally, and what to do and who to contact if the technology fails or they require help. Support for technological issues needs to be provided by the system itself, to avoid the responsibility falling on health care providers. These strategies will enhance patient reactions and responses to virtual care, which will result in better outcomes by increasing patient confidence in their ability to use virtual care and patient confidence in the health system’s ability to support them if the technology malfunctions or does not work as intended.

### Limitations

This study has certain limitations. First, while theory-driven research can provide transferable context-specific understandings of phenomena (in the case of this study, VFU for cancer), inferences about transferability must be tested. In other words, while we may assume that a CMOC operates in a different setting due to the presence of similar mechanisms, this claim should be tested using locally collected primary data. Any testing is aided by the fact that CMOCs are a form of middle-range theory and are expressed in a way that permits empirical testing using primary data. Though our sample was diverse in terms of race, ethnicity, first language, etc, the participants were predominantly high-income, highly educated, and highly activated individuals. As such, our findings may not fully represent the perspective of less activated or less educated patients who may have lower health literacy, be less involved in their care, and face more challenges with virtual care. It is possible that these individuals would differ in their satisfaction with VFU and its suitability as a method of follow-up care. This highlights a need for future research to better understand how VFU could be optimized for populations who face systemic and structural barriers to the use of virtual care. Finally, it may be a limitation that a small subset of participants (6/24, 25%) did not experience an in-person appointment within 12 months of participation. Though it is likely they received an in-person appointment before this date, it is possible that they lacked experience with in-person cancer care with which to compare virtual cancer care. However, we found no obvious differences in the findings between participants who did and did not report in-person appointments during the 12-month period.

### Conclusions

Patients value VFU as a part of their care; however, VFU is not working as well as it could for them. To optimize VFU, it is critical to consider the contextual factors and underlying mechanisms that influence patient perceptions of the patient centeredness and effectiveness of their care. Offering patients the choice of in-person, telephone, or video visits when possible, coupled with streamlined access to in-person care when required, is important. Prioritizing and addressing patient needs; enhancing physician online socioemotional behaviors and technology competency; and enhancing VFU functionality, usability, and processes of care and communication workflows will improve the patient centeredness of virtual care. By improving these contextual factors, VFU can be aligned with patient needs and preferences, resulting in effective, efficient, safe, and equitable follow-up care.

## Appendix

Multimedia Appendix 1 Conceptual buckets, themes, and context-mechanism-outcome configurations.

## Abbreviations

ASCO: American Association of Clinical Oncology
BC: breast cancer
CMOC: context-mechanism-outcome configuration
PC: prostate cancer
PM: Princess Margaret
VFU: virtual follow-up
