# Supplementary material for: Optimizing Virtual Follow-Up Care: Realist Evaluation of Experiences and Perspectives of Patients With Breast and Prostate Cancer
Source: J Med Internet Res. 2025 Jan 3;27:e65148. doi: 10.2196/65148 (PMC11748426; doi:10.2196/65148)
Supplement: Multimedia Appendix 1 [file jmir_v27i1e65148_app1.docx]

**Multimedia Appendix 1.** Conceptual buckets, themes, and context-mechanism-outcome configurations (CMOCs).

| Code^a^ | | | CMOC |
| --- | --- | --- | --- |
| **A. Effectiveness bucket** | | | |
|  | **Theme 1: loss of physical examinations** | | |
|  |  | A1 | When survivors of cancer believe that physical examination is an important part of their follow-up care (C^b^), they feel anxious (O^c^) because they fear disease progression or recurrence will be missed with VFU^d^ (M^e^) |
|  | **Theme 2: loss of visual cues** | | |
|  |  | A2 | When a patient has a follow-up appointment face-to-face (video or in person; C), they receive visual information from the care provider (cues, body language, and facial expressions) that helps them better understand what their care provider is saying (M), making them feel more reassured (O) |
|  |  | A3 | When a patient is anxious about their cancer (C), they prefer face-to-face consultation (in person or via video; O) because they find non–face-to-face consultations (ie, phone calls) insufficiently reassuring (M) |
|  |  | A4 | When a patient has a VFU appointment (by phone; C), they feel less confident in the quality of their care (O) because the clinician cannot use visual cues or body language to assess their mental well-being (ie, distress and concern; M) |
|  | **Theme 3: loss of appreciation of the seriousness of the condition** | | |
|  |  | A5 | When patients are no longer being seen in person regularly (C), they become less actively engaged in their care (O) because they lose the sense of the seriousness of their illness that in-person appointments provide (M) |
| **B. Efficiency and timeliness bucket** | | | |
|  | **Theme 1: improves health care system efficiency and capacity** | | |
|  |  | B1 | When physicians can see more patients virtually (vs face-to-face; C), patients feel it increases the efficiency of follow-up (O) because of the greater throughput (because more people can be seen; M) |
|  |  | B2 | When some patients are being followed up virtually (C) other patients are more satisfied with their care (O) because the waiting room is less busy and stressful (M) |
|  | **Theme 2: creates delays in accessing care** | | |
|  |  | B3 | When VFU does not happen as planned for the patient and they are not informed as to why (C), they are less satisfied with VFU (O) because they have been kept in the dark (M) |
|  |  | B4 | When VFU is delivered in such a way as to create additional barriers to meeting patients’ needs and expectations (C), they become frustrated (O) because of the inconvenience (M) |
|  |  | B5 | When patients have a health concern and VFU makes it harder to contact their physician (C), they feel anxious (O) because they fear their condition will worsen without timely care (M) |
| **C. Patient-centeredness bucket** | | | |
|  | **Theme 1: poorer continuity of care** | | |
|  |  | C1 | When there is a lack of continuity of care from the same care provider over time (C), patients are less satisfied with VFU (O) because they have not built a trusting relationship with their care provider (M) |
|  |  | C2 | When patients who have a new or recent cancer diagnosis see a greater number of care providers during VFU (C), they feel anxious (O) because they are particularly vulnerable, and VFU makes it more difficult to form trusting relationships (M) |
|  |  | C3 | When patients see a greater number of care providers during VFU and have to keep repeating their history (C), they are less satisfied with VFU (O) because they find this frustrating (M) |
|  |  | C4 | When patients see a greater number of care providers during VFU and physicians do not keep themselves up-to-date on a patient’s file (C), patients are less confident in the quality of their care (O) because they are concerned that the care providers do not have a comprehensive understanding of their history and case (M) |
|  | **Theme 2: lack of visual cues erodes connection and confidence** | | |
|  |  | C5 | When a patient has not met their care provider face-to-face (eg, in person or via video; C), it makes it difficult for them to build a trusting relationship with them (O) because of the lack of connection due to a lack of visual cues (M) |
|  |  | C6 | When a patient has a non-face-to-face follow-up appointment (ie, telephone; C), the patient cannot see that they have the full attention of their care provider (M), making them feel less confident in the quality of their care (O) |
|  | **Theme 3: feeling rushed** | | |
|  |  | C7 | When patients feel rushed or are not provided sufficient time to ask questions and bring up concerns during phone follow-up appointments (C), they feel less cared for (O) because they do not feel listened to (M) |
|  | **Theme 4: lack of emotional support** | | |
|  |  | C8 | When patients do not receive support for the emotional or personal concerns that accompany a cancer diagnosis during VFU (C), they are less satisfied (O) because their needs have not been met (M) |
|  | **Theme 5: feeling abandoned** | | |
|  |  | C9 | When health care professionals provide VFU (C), they tend to focus more on the information-giving aspects of the consultation (O) because virtual modality tends to favor information giving (M) |
|  |  | C10 | When patients complete treatment and enter into VFU (C), they may feel abandoned (O) because it is more difficult to contact their care provider and administrative support virtually (M) |
|  |  | C11 | When patients are provided information resources (ie, FAQs^f^, chatbots, and pamphlets) to answer simple questions that they could not ask during VFU appointments (ie, because they feel rushed; C), they feel less anxious (O) because they can get their questions answered in a timely manner (M) |
|  |  | C12 | When a patient has the option of communicating with their care provider via technology (ie, email, direct messaging through portal, and SMS text messages) between scheduled VFU visits and the care provider responds in a timely way (C), they feel less anxious (O) because they can get their questions answered in a more timely manner (M) |
|  |  | C13 | When clinicians do not spend time during VFU referring patients to supports/services and patients can no longer access these resources in person (ie, pamphlets, drop-in centers, and support groups; C), they may feel abandoned (O) because they have no idea of where to turn for support (M) |
|  | **Theme 6: improves access and efficiency of care** | | |
|  |  | C14 | When patients face barriers to accessing in-person appointments (C), they prefer VFU (O) because it is more convenient (M) |
|  | **Theme 7: more convenient** | | |
|  |  | C15 | Patients may experience less frustration (O) when waiting for a VFU appointment rather than waiting for an in-person appointment (C) because they can wait at a location of their choice and use the time to do other things (M) |
|  | **Theme 8: includes the family** | | |
|  |  | C16 | When supportive family members/partners are allowed to join in a VFU appointment (C), the patient can feel better supported (O) and the family member can feel better informed (O) because the family member is present to listen and ask questions (M) |
| **D. Equity bucket** | | | |
|  | **Theme 1: VFU does not work for everyone** | | |
|  |  | D1 | For patients who have specific communication difficulties (C), VFU may not be effective (O) because it is more difficult to understand the message because of their impairment (M) |
|  |  | D2 | For patients with poor quality internet (C), VFU may be effective (O) because the technology may not perform effectively (M) |
|  |  | D3 | For patients with lower eHealth literacy (C), VFU may not be effective (O) because they cannot find health-related information on the internet to fill in the gaps left by the lack of information received during the VFU appointment (M) |
| **E. Safety bucket** | | | |
|  | **Theme 1: turning elsewhere for care** | | |
|  |  | E1 | When patients cannot receive in-person care from their specialist and are forced to access care from nonspecialists (ie, emergency rooms; C), they may experience harm (O) because the care they receive may not be appropriate (M) |
|  | **Theme 2: avoiding exposure to infectious disease** | | |
|  |  | E2 | When patients are concerned about catching infectious diseases when being followed up face-to-face (C), they prefer VFU (O) because they believe it reduces their risk of exposure to infectious diseases (M) |
| **F. Patient characteristics bucket** | | | |
|  | **Theme 1: comfort with technology** | | |
|  |  | F1 | When people do not know how to use VFU technology (C), they are less willing to use it (O) because VFU makes them uncomfortable (M) |
|  |  | F2 | When an individual has had positive experiences using VFU technology (C), they are more willing to use it (O) because they can appreciate its value (M) |
|  | **Theme 2: disease characteristics** | | |
|  |  | F3 | When patients do not believe that their health problem can be adequately addressed by VFU (ie, lots of concerns about their cancer, complex cases, side effects, and new or recent cancer diagnosis; C), they are less satisfied with VFU (O) because they do not feel it sufficiently meets their needs (M) |
|  |  | F4 | When patients believe that their health problem can be adequately addressed by VFU (eg, simple routine matters such as medication renewals, providing normal test results, people in remission for some time, and people who have fewer needs; C), they are more likely to use it/be satisfied with it (O) because it is quick and convenient/sufficiently meets their needs (M) |
|  |  | F5 | When a patient is reluctant to access the health care system (C), they are more satisfied with VFU (O) because it is more efficient and convenient compared to in-person care (M) |
|  | **Theme 3: coping styles** | | |
|  |  | F6 | When patients who engage in problem-focused coping receive simple/quick VFU appointments (C), they are satisfied with VFU (O) because they place less value on/are less comfortable with/do not need the emotional aspects of care (M) |
|  |  | F7 | When patients who engage in emotional-focused coping receive simple/quick VFU appointments (C), they are less satisfied with VFU (O) because they require the emotional aspects of care (M) |
| **G. Provider characteristics bucket** | | | |
|  | **Theme 1: comfort with technology** | | |
|  |  | G1 | When physicians struggle with using the technology for VFU (C), it makes patients less confident in the quality of their care (O) because patients develop concerns about their medical competence (M) |
|  | **Theme 2: poor “webside” manner** | | |
|  |  | G2 | When physicians do not use “bedside manners” during VFU appointments (C), patients feel less cared for (O) because they perceive their care provider as less empathetic (M) |
|  |  | G3 | When physicians focus only on the information-giving aspect of follow-up care during VFU appointments (C), patients feel less cared for (O) because their emotional needs are not being met (M) |
|  |  | G4 | Patients believe that if providers received training on and practiced good webside manners (C), they would be more satisfied with VFU (O) because they would feel more cared for (M) |
| **H. Virtual care system characteristics bucket** | | | |
|  | **Theme 1: providing the option for video** | | |
|  |  | H1 | When patients are offered the option of having a video consultation (C), they are more satisfied with VFU (O) because it makes them feel respected (M) |
|  | **Theme 2: usability and functionality** | | |
|  |  | H2 | When VFU technology is not easy to use (C), patients are less willing to use it (O) because it causes frustration (M) |
|  |  | H3 | When patients have chosen to use VFU (C), providers must offer fallback plans for technical failures (O) because this allays patient concerns (M) |
|  | **Theme 3: establishing virtual process of care and communication channels** | | |
|  |  | H4 | If virtual processes of care and communication channels are not established (C), it will not work well (O) because patients are not receiving clear communication on what to expect (M) and they find it difficult to access support staff and schedule appointments (M) |

^a^Codes refer to which CMOCs relate to respective themes throughout the manuscript

^b^C: Context

^c^M: Mechanism

^d^VFU: virtual follow-up.

^e^O: Outcome

^f^FAQ: frequently asked question.
